# 5,5′-Dimethyl-2,2′-bipyridine

**DOI:** 10.1107/S1600536809019151

**Published:** 2009-06-17

**Authors:** Zeinab Khoshtarkib, Amin Ebadi, Roya Ahmadi, Robabeh Alizadeh

**Affiliations:** a Islamic Azad University, Shahr-e-Rey Branch, Tehran, Iran; b Department of Chemistry, Islamic Azad University, Kazerun Branch, Kazerun, Fars, Iran; c Damghan University of Basic Sciences, School of Chemistry, Damghan, Iran

## Abstract

The asymmetric unit of the title compound, C_12_H_12_N_2_, contains two half-mol­ecules related by an inversion center, the planes of their pyridine rings being oriented at a dihedral angle of 69.62 (4)°. In the crystal structure, a π–π contact between the pyridine rings [centroid–centroid distance = 3.895 (3) Å] may stabilize the structure. A weak C—H⋯π inter­action is also found.

## Related literature

For related structures, see: Ahmadi *et al.* (2008 ▶); Albada *et al.* (2004 ▶); Amani *et al.* (2007 ▶); Kalateh *et al.* (2008 ▶); Khalighi *et al.* (2008 ▶); Maheshwari *et al.* (2007 ▶); Tadayon Pour *et al.* (2008 ▶). For bond-length data, see: Allen *et al.* (1987 ▶).
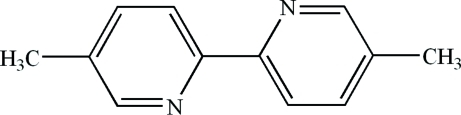


## Experimental

### 

#### Crystal data

C_12_H_12_N_2_
*M* *_r_* = 184.24
Triclinic, 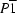

*a* = 6.409 (4) Å
*b* = 7.312 (5) Å
*c* = 11.533 (8) Å
α = 96.04 (5)°
β = 91.16 (4)°
γ = 105.03 (5)°
*V* = 518.4 (6) Å^3^
*Z* = 2
Mo *K*α radiation
μ = 0.07 mm^−1^
*T* = 298 K
0.50 × 0.41 × 0.29 mm

#### Data collection

Bruker SMART CCD area-detector diffractometer
Absorption correction: none
6191 measured reflections
2739 independent reflections
2067 reflections with *I* > 2σ(*I*)
*R*_int_ = 0.082

#### Refinement

*R*[*F* ^2^ > 2σ(*F* ^2^)] = 0.066
*wR*(*F* ^2^) = 0.205
*S* = 1.08
2739 reflections
129 parameters
H-atom parameters constrained
Δρ_max_ = 0.27 e Å^−3^
Δρ_min_ = −0.24 e Å^−3^

### 

Data collection: *SMART* (Bruker, 1998 ▶); cell refinement: *SAINT* (Bruker, 1998 ▶); data reduction: *SAINT*; program(s) used to solve structure: *SHELXTL* (Sheldrick, 2008 ▶); program(s) used to refine structure: *SHELXTL*; molecular graphics: *ORTEP-3 for Windows* (Farrugia, 1997 ▶); software used to prepare material for publication: *WinGX* (Farrugia, 1999 ▶).

## Supplementary Material

Crystal structure: contains datablocks I, global. DOI: 10.1107/S1600536809019151/hk2693sup1.cif

Structure factors: contains datablocks I. DOI: 10.1107/S1600536809019151/hk2693Isup2.hkl

Additional supplementary materials:  crystallographic information; 3D view; checkCIF report

## Figures and Tables

**Table 1:** Hydrogen-bond geometry (Å, °)

*D*—H⋯*A*	*D*—H	H⋯*A*	*D*⋯*A*	*D*—H⋯*A*
C9—H9⋯*Cg*1^i^	0.93	2.78	3.669 (3)	160

Symmetry code: (i) 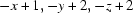. *Cg*1 is the centroid of the N1,C1–C4,C6 ring.

**Fig. 1.**
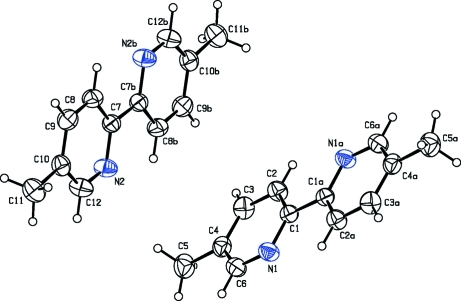
The molecular structure of the title molecule, with the atom-numbering scheme. Displacement ellipsoids are drawn at the 30% probability level [symmetry codes: (a) 1 - x, 2 - y, 1 - z, (b) -x, 2 - y, 2 - z].

**Fig. 2.**
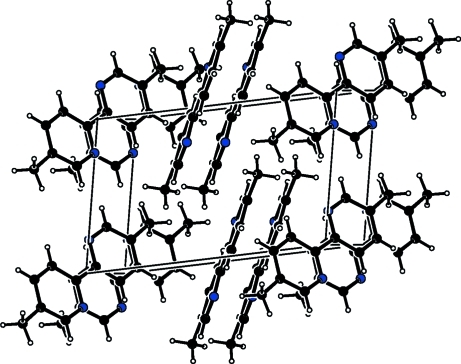
A partial packing diagram of the title compound.

## supplementary crystallographic information

### Comment

5,5'-Dimethyl-2,2'-bipyridine, (5,5'-dmbipy), is a good bidentate ligand, and numerous complexes with 5,5'-dmbipy have been prepared, such as that of zinc (Khalighi *et al.*, 2008), mercury (Tadayon Pour *et al.*, 2008), iron (Amani *et al.*, 2007), indium (Kalateh *et al.*, 2008), cadmium (Ahmadi *et al.*, 2008), copper (Albada *et al.*, 2004) and platin (Maheshwari *et al.*, 2007). We report herein the crystal structure of the title compound.

The asymmetric unit of the title compound (Fig.1) contains two halves molecules, in which the bond lengths (Allen *et al.*,  1987) and angles are within normal ranges. Rings A (N1/C1-C4/C6) and B (N2/C7-C10/C12) are, of course, planar and they are oriented at a dihedral angle of A/B = 69.62 (4)°.

In the crystal structure (Fig. 2), the π–π contact between the pyridine rings, Cg2—Cg2^i^ [symmetry code: (i) 1 - x, 2 - y, 2 - z, where Cg2 is centroid of the ring B (N2/C7-C10/C12)] may stabilize the structure, with a centroid-centroid distance of 3.895 (3) Å. There also exists a weak C—H···π interaction (Table 1).

### Experimental

For the preparation of the title compound, a solution of 5,5'-dimethyl-2,2' -bipyridine (0.15 g, 0.80 mmol) in methanol (15 ml) was added to a solution of BaCl_2_.2H_2_O, (0.10 g, 0.40 mmol) in water (5 ml) and the resulting colorless solution was stirred for 10 min at 313 K. Then, it was left to evaporate slowly at room temperature. After one week, colorless prismatic crystals of the title compound were isolated.

### Refinement

H atoms were positioned geometrically, with C-H = 0.93 and 0.96 Å for aromatic and methyl H, respectively, and constrained to ride on their parent atoms, with U_iso_(H) = 1.2U_eq_(C).

### Crystal data

**Table:**

C_12_H_12_N_2_	*Z* = 2
*M**_r_* = 184.24	*F*(000) = 196
Triclinic, *P*1	*D*_x_ = 1.180 Mg m^−^^3^
Hall symbol: -P 1	Mo *K*α radiation, λ = 0.71073 Å
*a* = 6.409 (4) Å	Cell parameters from 1133 reflections
*b* = 7.312 (5) Å	θ = 1.8–29.3°
*c* = 11.533 (8) Å	µ = 0.07 mm^−^^1^
α = 96.04 (5)°	*T* = 298 K
β = 91.16 (4)°	Prism, colorless
γ = 105.03 (5)°	0.50 × 0.41 × 0.29 mm
*V* = 518.4 (6) Å^3^	

### Data collection

**Table:**

Bruker SMART CCD area-detector diffractometer	2067 reflections with *I* > 2σ(*I*)
Radiation source: fine-focus sealed tube	*R*_int_ = 0.082
graphite	θ_max_ = 29.3°, θ_min_ = 1.8°
φ and ω scans	*h* = −8→8
6191 measured reflections	*k* = −10→10
2739 independent reflections	*l* = −15→15

### Refinement

**Table:**

Refinement on *F*^2^	Primary atom site location: structure-invariant direct methods
Least-squares matrix: full	Secondary atom site location: difference Fourier map
*R*[*F*^2^ > 2σ(*F*^2^)] = 0.066	Hydrogen site location: inferred from neighbouring sites
*wR*(*F*^2^) = 0.205	H-atom parameters constrained
*S* = 1.08	*w* = 1/[σ^2^(*F*_o_^2^) + (0.1057*P*)^2^ + 0.0566*P*] where *P* = (*F*_o_^2^ + 2*F*_c_^2^)/3
2739 reflections	(Δ/σ)_max_ = 0.002
129 parameters	Δρ_max_ = 0.27 e Å^−^^3^
0 restraints	Δρ_min_ = −0.24 e Å^−^^3^

### Special details

**Table:**

Geometry. All e.s.d.'s (except the e.s.d. in the dihedral angle between two l.s. planes) are estimated using the full covariance matrix. The cell e.s.d.'s are taken into account individually in the estimation of e.s.d.'s in distances, angles and torsion angles; correlations between e.s.d.'s in cell parameters are only used when they are defined by crystal symmetry. An approximate (isotropic) treatment of cell e.s.d.'s is used for estimating e.s.d.'s involving l.s. planes.
Refinement. Refinement of *F*^2^ against ALL reflections. The weighted *R*-factor *wR* and goodness of fit *S* are based on *F*^2^, conventional *R*-factors *R* are based on *F*, with *F* set to zero for negative *F*^2^. The threshold expression of *F*^2^ > σ(*F*^2^) is used only for calculating *R*-factors(gt) *etc*. and is not relevant to the choice of reflections for refinement. *R*-factors based on *F*^2^ are statistically about twice as large as those based on *F*, and *R*- factors based on ALL data will be even larger.

### Fractional atomic coordinates and isotropic or equivalent isotropic displacement parameters (Å^2^)

**Table:**

	*x*	*y*	*z*	*U*_iso_*/*U*_eq_	
N1	0.4778 (2)	0.76393 (19)	0.53822 (13)	0.0604 (4)	
N2	0.0741 (2)	0.77876 (19)	0.99037 (14)	0.0656 (4)	
C1	0.4223 (2)	0.92544 (19)	0.52837 (12)	0.0486 (3)	
C2	0.2280 (2)	0.9529 (2)	0.56860 (14)	0.0579 (4)	
H2	0.1916	1.0661	0.5601	0.069*	
C3	0.0896 (3)	0.8105 (3)	0.62124 (16)	0.0655 (4)	
H3	−0.0407	0.8276	0.6480	0.079*	
C4	0.1445 (3)	0.6443 (2)	0.63387 (13)	0.0594 (4)	
C5	0.0011 (4)	0.4859 (3)	0.69263 (18)	0.0797 (6)	
H5C	0.0475	0.3716	0.6761	0.096*	
H5B	−0.1459	0.4638	0.6637	0.096*	
H5A	0.0105	0.5218	0.7755	0.096*	
C6	0.3403 (3)	0.6293 (2)	0.58998 (16)	0.0641 (4)	
H6	0.3793	0.5168	0.5971	0.077*	
C7	0.0828 (2)	0.96057 (19)	1.02576 (12)	0.0513 (4)	
C8	0.2406 (3)	1.0695 (2)	1.10747 (14)	0.0632 (4)	
H8	0.2442	1.1958	1.1315	0.076*	
C9	0.3905 (3)	0.9892 (3)	1.15227 (15)	0.0679 (5)	
H9	0.4973	1.0614	1.2066	0.082*	
C10	0.3838 (3)	0.8018 (2)	1.11714 (14)	0.0600 (4)	
C11	0.5426 (3)	0.7065 (3)	1.1636 (2)	0.0793 (5)	
H11C	0.5070	0.5755	1.1305	0.095*	
H11B	0.5374	0.7130	1.2470	0.095*	
H11A	0.6856	0.7699	1.1431	0.095*	
C12	0.2214 (3)	0.7049 (2)	1.03580 (18)	0.0686 (5)	
H12	0.2146	0.5782	1.0109	0.082*	

### Atomic displacement parameters (Å^2^)

**Table:**

	*U*^11^	*U*^22^	*U*^33^	*U*^12^	*U*^13^	*U*^23^
N1	0.0614 (8)	0.0537 (7)	0.0719 (8)	0.0235 (6)	0.0113 (6)	0.0111 (6)
N2	0.0615 (8)	0.0483 (7)	0.0819 (9)	0.0103 (6)	−0.0066 (7)	−0.0034 (6)
C1	0.0470 (7)	0.0513 (7)	0.0491 (7)	0.0170 (6)	−0.0020 (5)	0.0031 (5)
C2	0.0500 (8)	0.0626 (9)	0.0669 (9)	0.0231 (6)	0.0037 (6)	0.0129 (7)
C3	0.0508 (8)	0.0790 (11)	0.0696 (9)	0.0200 (8)	0.0091 (7)	0.0131 (8)
C4	0.0607 (9)	0.0615 (9)	0.0513 (7)	0.0076 (7)	0.0001 (6)	0.0067 (6)
C5	0.0809 (13)	0.0784 (12)	0.0723 (11)	0.0034 (10)	0.0095 (9)	0.0176 (9)
C6	0.0734 (10)	0.0539 (8)	0.0690 (9)	0.0212 (7)	0.0104 (8)	0.0125 (7)
C7	0.0527 (7)	0.0454 (7)	0.0523 (7)	0.0062 (6)	0.0075 (6)	0.0052 (5)
C8	0.0722 (10)	0.0521 (8)	0.0608 (8)	0.0129 (7)	−0.0068 (7)	−0.0031 (6)
C9	0.0727 (10)	0.0664 (10)	0.0595 (9)	0.0131 (8)	−0.0108 (7)	0.0003 (7)
C10	0.0567 (8)	0.0625 (9)	0.0620 (8)	0.0137 (7)	0.0086 (7)	0.0158 (7)
C11	0.0705 (11)	0.0809 (13)	0.0911 (13)	0.0247 (10)	−0.0004 (10)	0.0194 (10)
C12	0.0640 (10)	0.0500 (8)	0.0896 (12)	0.0137 (7)	−0.0015 (8)	0.0017 (8)

### Geometric parameters (Å, °)

**Table:**

C1—N1	1.334 (2)	C7—N2	1.335 (2)
C1—C2	1.393 (2)	C7—C8	1.389 (2)
C1—C1^i^	1.491 (3)	C7—C7^ii^	1.475 (3)
C2—C3	1.384 (3)	C8—C9	1.368 (3)
C2—H2	0.9300	C8—H8	0.9300
C3—C4	1.371 (3)	C9—C10	1.377 (3)
C3—H3	0.9300	C9—H9	0.9300
C4—C6	1.389 (3)	C10—C12	1.380 (3)
C4—C5	1.511 (3)	C10—C11	1.494 (3)
C5—H5C	0.9600	C11—H11C	0.9600
C5—H5B	0.9600	C11—H11B	0.9600
C5—H5A	0.9600	C11—H11A	0.9600
C6—N1	1.340 (2)	C12—N2	1.327 (2)
C6—H6	0.9300	C12—H12	0.9300
			
N1—C1—C2	121.57 (15)	N2—C7—C7^ii^	116.86 (16)
N1—C1—C1^i^	116.75 (16)	C8—C7—C7^ii^	121.84 (17)
C2—C1—C1^i^	121.68 (16)	C9—C8—C7	119.40 (16)
C3—C2—C1	119.51 (15)	C9—C8—H8	120.3
C3—C2—H2	120.2	C7—C8—H8	120.3
C1—C2—H2	120.2	C8—C9—C10	120.16 (16)
C4—C3—C2	119.99 (16)	C8—C9—H9	119.9
C4—C3—H3	120.0	C10—C9—H9	119.9
C2—C3—H3	120.0	C9—C10—C12	116.32 (17)
C3—C4—C6	116.29 (16)	C9—C10—C11	122.45 (18)
C3—C4—C5	122.09 (17)	C12—C10—C11	121.22 (17)
C6—C4—C5	121.62 (17)	C10—C11—H11C	109.5
C4—C5—H5C	109.5	C10—C11—H11B	109.5
C4—C5—H5B	109.5	H11C—C11—H11B	109.5
H5C—C5—H5B	109.5	C10—C11—H11A	109.5
C4—C5—H5A	109.5	H11C—C11—H11A	109.5
H5C—C5—H5A	109.5	H11B—C11—H11A	109.5
H5B—C5—H5A	109.5	N2—C12—C10	124.98 (16)
N1—C6—C4	125.26 (16)	N2—C12—H12	117.5
N1—C6—H6	117.4	C10—C12—H12	117.5
C4—C6—H6	117.4	C1—N1—C6	117.36 (15)
N2—C7—C8	121.30 (15)	C12—N2—C7	117.84 (15)
			
N1—C1—C2—C3	0.7 (2)	C8—C9—C10—C12	0.4 (3)
C1^i^—C1—C2—C3	−179.66 (16)	C8—C9—C10—C11	−179.67 (17)
C1—C2—C3—C4	0.3 (3)	C9—C10—C12—N2	−0.2 (3)
C2—C3—C4—C6	−0.9 (3)	C11—C10—C12—N2	179.92 (17)
C2—C3—C4—C5	178.87 (16)	C2—C1—N1—C6	−0.9 (2)
C3—C4—C6—N1	0.7 (3)	C1^i^—C1—N1—C6	179.44 (15)
C5—C4—C6—N1	−179.06 (16)	C4—C6—N1—C1	0.2 (3)
N2—C7—C8—C9	0.2 (3)	C10—C12—N2—C7	−0.1 (3)
C7^ii^—C7—C8—C9	−179.88 (17)	C8—C7—N2—C12	0.0 (3)
C7—C8—C9—C10	−0.5 (3)	C7^ii^—C7—N2—C12	−179.87 (17)

Symmetry codes: (i) −*x*+1, −*y*+2, −*z*+1; (ii) −*x*, −*y*+2, −*z*+2.

### Hydrogen-bond geometry (Å, °)

**Table:**

*D*—H···*A*	*D*—H	H···*A*	*D*···*A*	*D*—H···*A*
C9—H9···Cg1^iii^	0.93	2.78	3.669 (3)	160

Symmetry codes: (iii) −*x*+1, −*y*+2, −*z*+2.
